# Conservation Planning for Ecosystem Services

**DOI:** 10.1371/journal.pbio.0040379

**Published:** 2006-10-31

**Authors:** Kai M. A Chan, M. Rebecca Shaw, David R Cameron, Emma C Underwood, Gretchen C Daily

**Affiliations:** 1 Center for Conservation Biology, Department of Biological Sciences, Stanford University, Stanford, California, United States of America; 2 The Nature Conservancy, San Francisco, California, United States of America; 3 Department of Environmental Science and Policy, University of California Davis, Davis, California, United States of America; The David and Lucile Packard Foundation, United States of America

## Abstract

Despite increasing attention to the human dimension of conservation projects, a rigorous, systematic methodology for planning for ecosystem services has not been developed. This is in part because flows of ecosystem services remain poorly characterized at local-to-regional scales, and their protection has not generally been made a priority. We used a spatially explicit conservation planning framework to explore the trade-offs and opportunities for aligning conservation goals for biodiversity with six ecosystem services (carbon storage, flood control, forage production, outdoor recreation, crop pollination, and water provision) in the Central Coast ecoregion of California, United States. We found weak positive and some weak negative associations between the priority areas for biodiversity conservation and the flows of the six ecosystem services across the ecoregion. Excluding the two agriculture-focused services—crop pollination and forage production—eliminates all negative correlations. We compared the degree to which four contrasting conservation network designs protect biodiversity and the flow of the six services. We found that biodiversity conservation protects substantial collateral flows of services. Targeting ecosystem services directly can meet the multiple ecosystem services and biodiversity goals more efficiently but cannot substitute for targeted biodiversity protection (biodiversity losses of 44% relative to targeting biodiversity alone). Strategically targeting only biodiversity plus the four positively associated services offers much promise (relative biodiversity losses of 7%). Here we present an initial analytical framework for integrating biodiversity and ecosystem services in conservation planning and illustrate its application. We found that although there are important potential trade-offs between conservation for biodiversity and for ecosystem services, a systematic planning framework offers scope for identifying valuable synergies.

## Abstract 2

A spatially explicit framework reveals whether areas important for the provision of ecosystem services align with areas important for biodiversity conservation and indicates the need to broaden current conservation goals.

## Introduction

Despite the vital importance of ecosystem services—the supply of benefits from ecosystems to society—leaders in both the private and public sectors have been slow to incorporate these benefits into decision making. This slow incorporation traces to a complex of factors well beyond science, but at the core is the poor characterization of the flow of services in the necessary biophysical and economic terms at the local and regional scales most useful to decision makers [1–3]. In recent years, however, there have been tremendous advances in the science, economic valuation, institutional design, and social capacity needed for ecosystem-service conservation.

These advances have come on numerous fronts, including the following: (i) the maturation of conservation planning [4–7]; (ii) the Millennium Ecosystem Assessment [8,9] and related work on particular services such as crop pollination [10–13], the regulation of water flow and hydropower production [14], the provision of water quality and quantity [15], and recreation [16]; (iii) the application of economic valuation techniques to ecosystem services [17–21]; (iv) the refinement of quantitative approaches and tools for decision-making [22–29]; and (v) institutional and other social change for ecosystem-service protection [30–32]. Yet, perhaps the most important and certainly the most innovative advances in ecosystem-service protection come from the emergence worldwide of small-scale systems of payment for ecosystem services [33–35].

Replicating and scaling up these small-scale efforts is a challenge requiring formal strategic planning for ecosystem services and biodiversity. To begin to address this challenge, we applied spatially explicit biodiversity-planning methodologies to a suite of six ecosystem services, yielding ecosystem-service conservation blueprints. We compared these to existing biodiversity blueprints to gain a preliminary understanding of trade-offs and side benefits between biodiversity and ecosystem services.

In developing a blueprint for biodiversity conservation, species, communities, and ecosystems are “features” for which “targets” are expressed [36]. The blueprint specifies protection of a “network” of specific places to meet the quantitative targets for these features [7]. There is no analog for most ecosystem services.

Planning for services is inherently more complex. The Millennium Ecosystem Assessment [8] distinguishes four categories of ecosystem services: provisioning (e.g., of seafood, timber), regulating (e.g., of climate, floods), supporting (of other services, e.g., pollination and pest control for food production), and cultural (e.g., serenity, inspiration). Daily et al. [37] also recognize the conservation of options (e.g., genetic diversity for future use). The key feature that distinguishes these services from ecosystem functions or processes is the explicit involvement of beneficiaries. As such, a proper characterization of ecosystem-service targets involves consideration of the demand for services—its magnitude and spatial distribution—in addition to the underlying ecosystem processes. For conservation nongovernmental organizations considering targeting ecosystem services, the alignment between service provision and biodiversity protection is critical. Although there has been some analysis of the provision of multiple ecosystem services simultaneously, these studies have generally been conducted at coarse spatial scales, not included space explicitly, or not accounted for variation in demand for services [31,38–47].

This paper has two parts. First, we demonstrate how a formal, quantitative planning framework can integrate ecosystem-service values into biodiversity conservation planning. Second, we provide a preliminary assessment of the possibilities for aligning goals of conserving biodiversity and ecosystem services. We adapted and applied methodologies for conservation planning to six ecosystem services of the Central Coast ecoregion in California, United States. We chose an area and services for which we had sufficient spatial data and an empirical understanding for an illustrative integrated analysis, rather than a definitive study of each service. The six ecosystem services—which are important locally, regionally, or globally—are described as follows.

Carbon storage is the carbon locked up in above- and below-ground biomass of primary producers. When natural vegetation cover is converted to agriculture or urban land, carbon is released to the atmosphere as carbon dioxide, exacerbating climate change [48]. Since 1850, more than a third of anthropogenic CO_2_ emissions has resulted from land conversion [49]. Accordingly, intact ecosystems provide a service to the global population by storing carbon.

Crop pollination is the pollination of crops by natural pollinators. Between 15% and 30% of the US food supply depends on animal-mediated pollination [50], and it is likely that many insect species other than the widely cultivated European honeybee contribute importantly to numerous crops [10]. Accordingly, pollinators from natural habitats provide a service to local food producers that is also critically important to humanity [51].

Flood control is the mitigation of flood risk, which is damaging to areas of agriculture and human settlement, that is mediated by land cover. Land-use change is an important contributor to increasing vulnerability to floods [52], and maintaining the right configuration of natural cover could result in savings of billions of dollars in damages [53].

Forage production is the production of forage for grazing rangeland livestock, an important economic land use in the grasslands and oak savannahs of this ecoregion. As a side benefit, livestock grazing may reduce the diversity of invasive species at fine scales [54,55], increase species richness and grassland diversity [55–57], and replicate disturbance processes previously generated by native herbivores [56].

Outdoor recreation is the provision of recreation opportunities by natural and semi-natural landscapes. Such outdoor activities are critically important to the economy of the Central Coast [58,59] and to the well-being of its residents.

Water provision is the supply of fresh water to meet the demand of the agricultural, industrial, and residential sectors of the Central Coast economy. Maintaining the flow of this service requires both limiting the degradation of water quality from agriculture and urban development and maintaining the active purification of water in wetlands and other habitats [60–63]. The quantity of this critical ecosystem service is in direct proportion to the amount of useable water that is available for human use.

With these six ecosystem services, we address the following questions: (i) How much of each service is being generated by each land parcel? (ii) What are the spatial associations between the lands required for protecting biodiversity and supplying different ecosystem services? (iii) How much of each ecosystem service—plus biodiversity protection—is provided by a network of lands prioritized for biodiversity, compared to networks designed for multiple services? By looking beyond benefits that can yield private gains, we are not limited by present market failures and thus broaden considerably the scope for ecosystem services provision to yield gains for conservation [64]. We follow the approach of Davis et al. [65] by avoiding the most problematic valuation methods (e.g., contingent valuation) and the difficult question of a common currency for disparate preferences and principles.

## Methods

**Figure 1:**
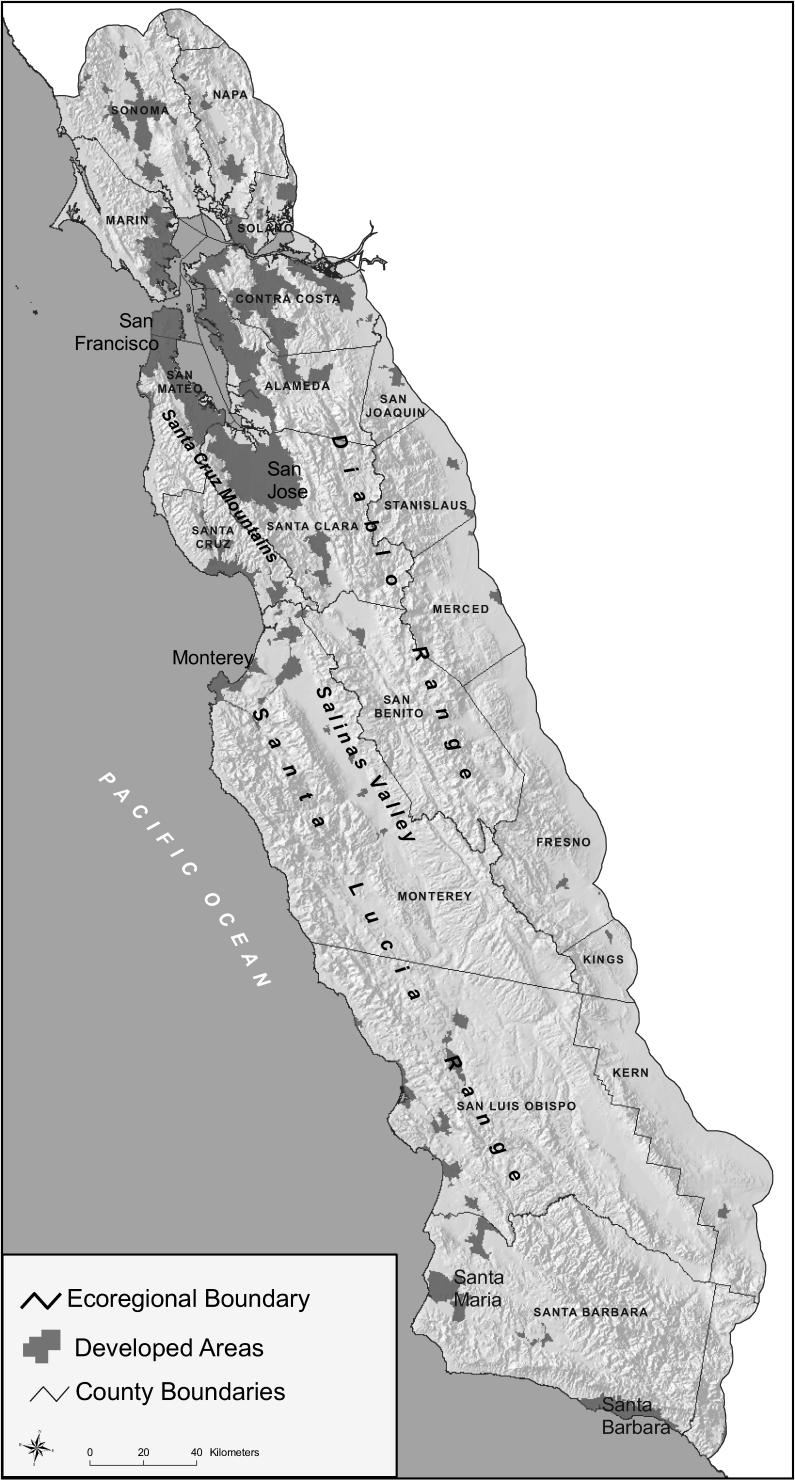
The Central Coast Ecoregion of California, with Geographic Features Mentioned in the Text

We focused on the Central Coast ecoregion of California (Figure 1), a geographically diverse area with steep climatic and edaphic gradients resulting in high plant diversity. The majority of the ecoregion's >9 million people reside within the San Francisco Bay area.

We characterized and mapped terrestrial biodiversity and the six ecosystem services listed above to develop networks of conservation areas for each service. We assembled these networks using MARXAN v1.8.2, an optimization algorithm [66,67]. MARXAN was developed to find systems of spatially cohesive sites that efficiently meet a suite of biodiversity targets.

We selected MARXAN to explore the mapping of ecosystem services for two reasons. First, this tool is used widely by conservation organizations to develop networks for biodiversity protection and is therefore the obvious tool to explore the mapping of other ecosystem services. Second, we wanted to compare the outcome of the planning process (prioritized areas) for each ecosystem service with that for biodiversity. We followed standard methods for ecoregional planning by The Nature Conservancy in creating the inputs necessary to assemble networks within MARXAN [6,68,69]. The key steps are described below, with specific details for each service provided in Table 1 and in Protocol S1.

**Table 1:**
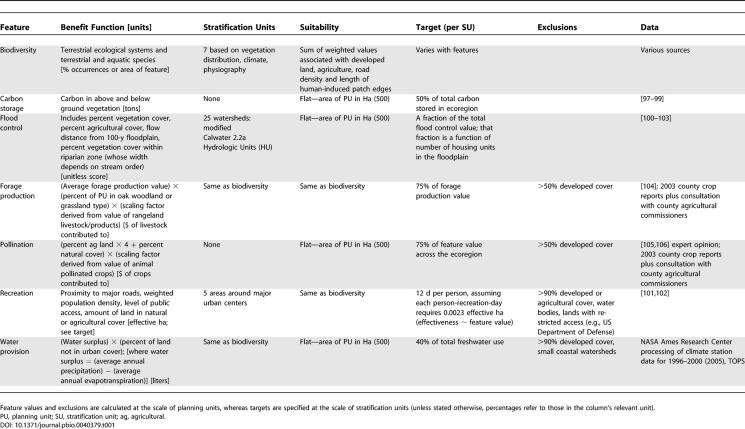
Details for Characterization and Prioritization in Site-Selection Software (MARXAN) of Biodiversity and the Six Ecosystem Services

Biodiversity conservation planning using MARXAN typically involves developing four sets of input variables. First: feature definition and mapping, where one identifies “features,” a subset of species and natural systems that represents the biodiversity of the area. These features are allocated by “planning units,” which are the uniform spatial unit of analysis (500 ha in this analysis). The “feature value” for each planning unit is the numerical value associated with a feature in that unit. Second: stratification, where one divides the study area into subregions to stratify feature occurrences across the full range of environmental gradients within the region. This ensures that features are captured across their range of environmental and genetic variation and also provides sufficient replication for persistence in the face of environmental change. Third: targets, where one sets targets to specify the quantities at which features should be represented in the network. These targets serve as initial hypotheses for testing the necessary levels of replication and abundance to ensure feature persistence. Targets are expressed as amount of viable occurrences or area within each stratification unit. Finally: suitability, where one defines the “suitability” of areas for conservation through numerical values that represent the degree of impediments to conservation success (also referred to as “costs”); This information is used to distribute conservation priorities to locations amenable to long-term persistence of features. Suitability defines the current degree of landscape degradation and fragmentation and/or the probability of degradation and fragmentation in the future; it usually comprises spatial data that represent current or future human infrastructure, activity, and land use. The suitability layer used in this analysis is described in Protocol S1.

With all the input variables in place, one runs MARXAN to select priority conservation areas that collectively constitute a network within the ecoregion. Because the complexity of the problems renders it impracticable to find exact analytical solutions, MARXAN assembles a network by randomly adding and removing planning units to maximize the coverage of features and their suitability. In this analysis, we ran MARXAN 20 times for each service to ensure an even sampling of possible networks. The “best” solution is the network that optimized the targets and maximized suitability; these are the networks discussed throughout unless specified otherwise. One can get a measure of the irreplaceability [70,71] of a planning unit from the number of times that it occurs in the full set of 20 networks, or the “summed-solution” network.

For the six ecosystem services, we altered the above steps to accommodate inherent differences in ecosystem services versus biodiversity. First, we created feature values by defining a benefit function, a function that expresses feature values (service production) at the scale of our planning units (500 ha). Our simple models capture the core factors influencing the relative production of each service across the ecoregion; they are sufficient to illustrate the framework and spatial relationships between services, but they are not adequate for a formal conservation plan that would give geographic direction to an organization's activities. Second, we attempted to distribute prioritized sites across the region through stratification units relevant to each service. Each stratification unit has its own target, which serves to limit the clustering of extractive impact (forage production) or to align service provision spatially with demand. Third, we set targets as percentages of total service produced within the ecoregion or, where possible, as quantities that reflect the local or regional (but not global) demand. Fourth, we used suitability data that reflected impediments to management; suitability layers were either the same as those for biodiversity (including a patch boundary-length term that encourages clustering of selected sites to better protect key ecological processes; Protocol S1) or a linear function of total area. Below we briefly introduce our models for each of the services, but refer readers interested in the details to Protocol S1.

**Carbon storage**: We estimated the amount of carbon stored by the above- and below-ground biomass in the ecoregion. We focused on storage rather than sequestration because of the considerable uncertainty regarding sequestration [72–75] and the importance of preventing the loss of stored carbon.

**Crop pollination**: We stipulated the value of this service to vary with the value of agricultural crops benefiting from animal pollination and with the presence of patches of natural vegetation to house those pollinators. Because even relatively small patches (smaller than our planning units) can provide important pollination benefits [10,12], we assumed that it would be possible to provide natural crop pollination even in planning units currently dominated by agriculture.

**Flood control**: Our model attempts to identify the areas important to maintain a natural flooding regime and reduce the risk of extreme flood events attributable to impervious surfaces in a watershed. The feature value of a planning unit varies with land cover in various categories and distance to the floodplain to reflect the flood-mitigation contribution of vegetation in floodplains [76,77], wetlands [78], the riparian zone [79,80], and even beyond [81,82].

**Forage production**: We modeled the value of forage production in an area as a function of climate, primary production of forage species, nutritional content of the forage, ability to withstand grazing, and the ability of ranchers to capture forage value through sales of livestock and livestock products.

**Outdoor recreation**: We estimated the value of recreation in an area as a function of the amount of natural and semi-natural habitat, and the accessibility of the area as measured by its proximity to population centers and major roads, as well as by the rights to access, as indicated by management designation.

**Water provision**: Our model for water provision attempts to quantify the amount of surplus clean water potentially available for human consumption. We represent water provision simply as precipitation minus evapotranspiration. We did not address built infrastructure for storing and distributing water, as we sought a simple way to account for the annual input into the ecoregion's surface reserves. Moreover, we did not distinguish between surface water and groundwater for several reasons: total water availability is limiting in much of the ecoregion; surface water and groundwater are connected environmentally in complex ways [83]; and users who cannot obtain sufficient water from one source will likely switch to the other.

### Analysis of Ecosystem Service Correlations and Overlap

To evaluate the spatial correspondence of biodiversity and the provision of services, we performed two types of tests: service correlation and network overlap. For service correlation, we calculated correlations (Pearson's *r*) between static feature values across all 11,272 planning units in the ecoregion. For comparison of correlations between biodiversity feature values with the other ecosystem services, we needed to distill the biodiversity value of a planning unit to a single number. To most closely parallel the six ecosystem services, we used the rarity-weighted richness index [RWRI, 84], which weights each feature at a site by the inverse of the number of planning units in which it occurs, so that rare features are weighted more heavily. These weightings are then summed across the features that occur in a planning unit.

The correlation between services does not fully convey the extent to which conservation activities for these services would align. First, correlations at low levels of provision are of limited relevance for prioritization, as planning units providing such service levels are unlikely to be selected for management. Feature correlations also neglect the spatial distributions of units necessary to meet conservation targets and the suitability for conservation. Accordingly, the overlap between selected sites (which account for both suitability and targets) is somewhat more relevant for conservation, and the comparison of correlation and overlap provides information regarding the influence of targets, suitability, and other factors.

We assessed network overlap in two ways: “best” network overlap and irreplaceability similarity. For the best network overlap, we calculated the number of cells for each pair-wise combination of services that occur in each best network and the number that co-occur. We calculated the expected number of overlapping cells based on an assumption of independence between the two networks as

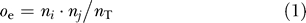
where *n_i_* is the number of planning units in the network associated with service *i*, *n_j_* is the number associated with service *j*, and *n_T_* is the total number of units (11,272). We considered two functions of these variables: the ratio of observed (*o*_o_) to expected numbers of overlapping cells (*o*_o_/*o*_e_), which determines the statistical significance; and the number of overlapping cells as a fraction of the number of cells in the smaller network [*o*_e_/min(*n_i_*,*n_j_*)], which determines practical significance as the proportion of the networks affected by these associations between services.

Because our interest is primarily in practical significance, and even associations with little practical importance will be statistically significant with such large sample sizes, we do not calculate statistical significance with sophistication and ignore the issues of spatial autocorrelation and multiple tests. For irreplaceability similarity, we calculated the correlation (Pearson's *r*) between the “summed-solution” networks (see above; because these results were very similar to overlaps in the optimal network, they are not shown).

### Strategies and Target Achievement

To illustrate the implications of expanding a conservation plan to include ecosystem services as well as biodiversity, we examined the trade-offs between biodiversity and ecosystem services goals and the potential added benefits of pursuing ecosystem services in addition to biodiversity (side benefits). For trade-offs, we analyzed four different combined networks of services: Biodiversity (only biodiversity); Non-biodiversity (all except biodiversity); All; and Strategic (using biodiversity, carbon storage, flood control, recreation, and water provision—i.e., excluding forage production and crop pollination that were characterized by negative associations with biodiversity).

Our “Strategic” network is intended to be strategic only in the context of this analysis to avoid trying to optimize across too many poorly correlated ends, because we recognize that many other factors must be considered in determining the course of conservation action. For “All” and “Strategic,” we used the same suitability layer as for “Biodiversity” and set MARXAN's cost threshold value to the same as that of the best “Biodiversity” run to ensure that these networks would be comparable. For “Non-biodiversity,” we used a flat cost value of 500 for each planning unit. Otherwise, the same MARXAN parameters were used as those described in the Protocol S1 for the individual scenarios.

For each combined network, we assessed the proportion of ecosystem-service targets achieved for each stratification unit (feature value captured/amount of feature value in target). We capped these target-achievement proportions at 1, because exceeding one stratification unit's target does not easily compensate for failing to meet another stratification unit's target. We calculated the ecoregion-wide target achievements for each ecosystem service by averaging across stratification units weighted by the size of the target. In some cases, it will be advantageous to exceed targets, however; accordingly, we separately calculated surpluses (max(0, feature value – target)/target) for each stratification unit and for the ecoregion (again through weighted averages).

For side-benefit analyses, we performed MARXAN runs to meet individual ecosystem-service targets assuming prior protection of the biodiversity network. We calculated the additional land needed (as a percentage of the biodiversity network), the additional “cost” of this land (using the service's suitability layer), and the benefit of these added lands for biodiversity (using RWRI).

## Results/Discussion

**Figure 2:**
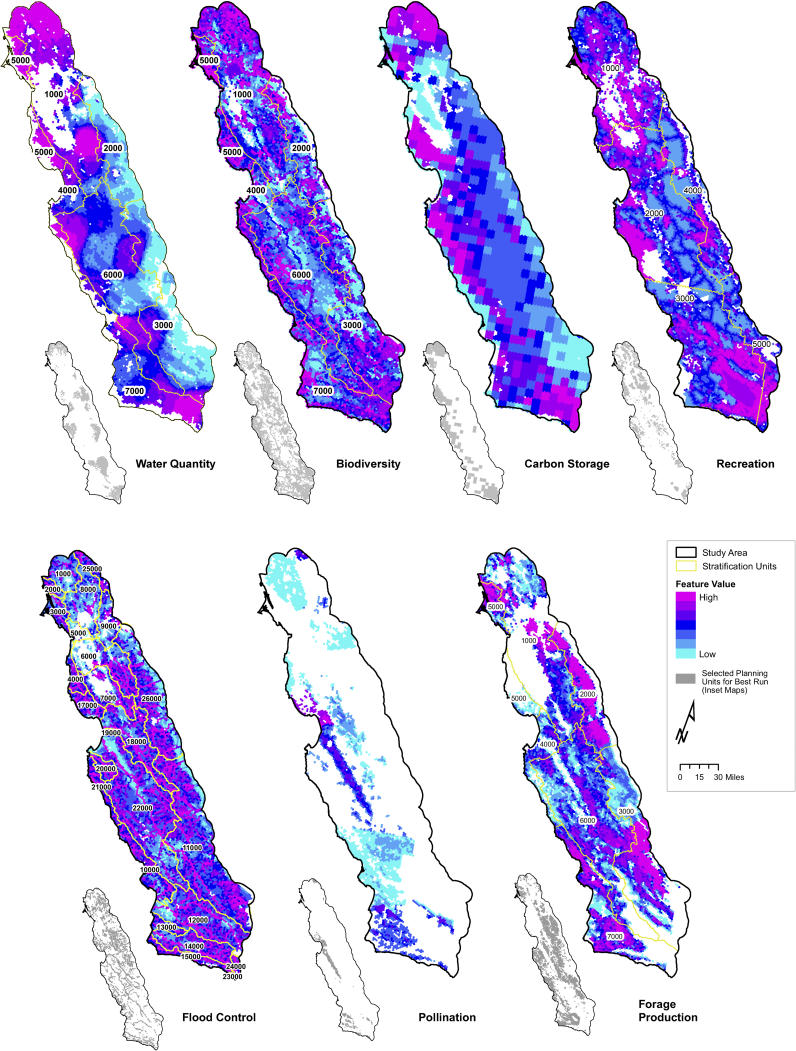
Spatial Analysis of Biodiversity and the Chosen Ecosystem Services The seven benefit functions (feature values) are displayed in color with the accompanying best networks of selected planning units in gray insets. Feature values range from 0 (or locked out; white), to low (light blue), moderate (dark blue), and high (purple). The boundary indicates the ecoregion plus the 10-km buffer. Yellow lines indicate stratification units, within which individual targets were pursued. Numbers in the thousands (×000) are stratification unit labels. Not shown are planning-unit–specific constraints and stratification-unit–specific targets.

The seven benefit functions—for biodiversity, carbon storage, flood control, forage production, pollination, recreation, and water provision—have distinctly different spatial distributions, although some areas are of high value to multiple services and other areas are of low value to many (Figure 2). For example, the largest agricultural valley in the ecoregion, Salinas Valley, is characterized by the following: a wide swath of high-value pollination services driven by the high proportion of land under crops benefiting from animal pollination; a narrow area of high–flood control services due to riparian vegetation; and low values of other services. Similarly, the mountain ranges throughout the ecoregion are characterized by natural forest cover (accounting for carbon storage values), high precipitation (water provision), and proximity to major population centers and accessibility by road (recreation). Accordingly, the following areas share high values for carbon storage, recreation, and water provision: the Santa Cruz Mountains, the Santa Lucia Mountains along the Big Sur Coast, and the northern Diablo Range.

**Figure 3:**
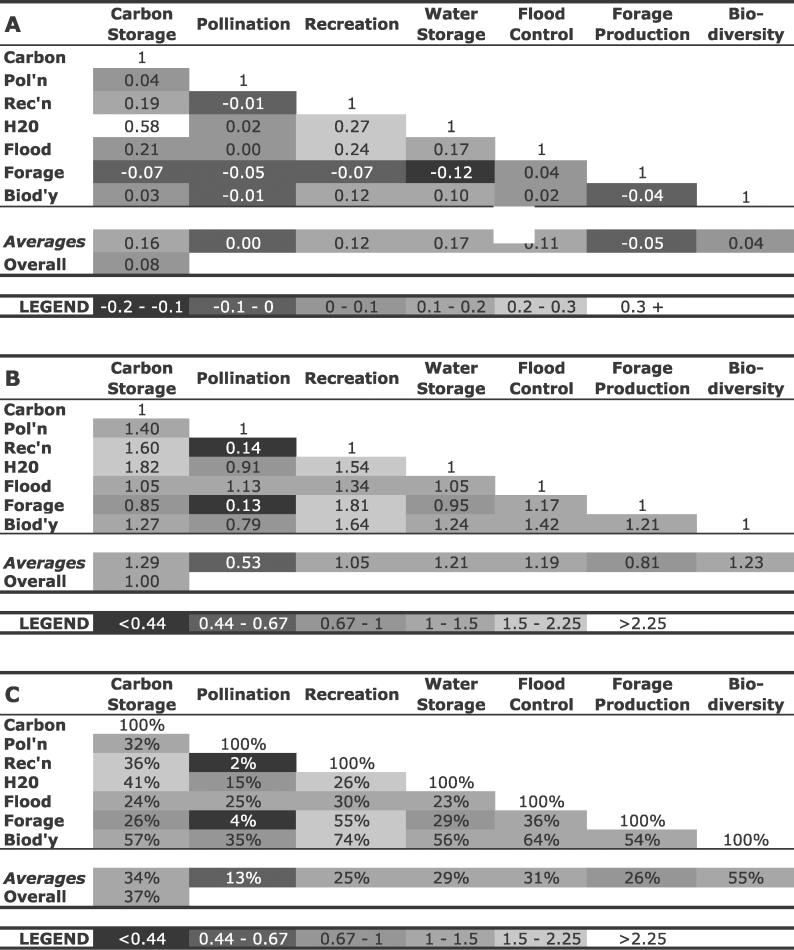
Pair-Wise Spatial Associations between Biodiversity and the Production of Ecosystem Services Associations are expressed as (A) the correlation coefficient (*r*) between feature values; (B) actual/expected number of planning units shared between best networks; and (C) actual number of shared planning units as a percentage of the smaller network. Arithmetic averages for each ecosystem service with each other ecosystem service are also noted. Shading indicates strength of correlation/overlap, as indicated by the legends. The shading in (C) follows values in (B). All correlations are statistically significant except for pollination with recreation, flood control, and biodiversity. All overlaps are statistically significant by *G*-tests for goodness of fit.

The spatial correlations between the ecosystems services are low (Figure 3A), with nearly as many negative correlations as positive ones. The overall average correlation is positive but low (0.08). The average correlation between biodiversity and services is also low. The highest correlation is between carbon storage and water provision (0.58). Other relatively high correlations (>0.2) are between recreation and water provision (reflecting the importance of natural cover to both) and between recreation and flood control. The latter correlation reflects the combination of the benefits of natural cover, the accessibility of riparian areas by road (recreation), and the importance of those areas for flood control. Although riparian areas are also important for water quality and aquatic diversity, neither of these was treated explicitly, so the value of riparian areas is underrepresented in our analysis. Negative correlations are restricted to pollination and forage production with other features.

The pair-wise overlap between the seven networks derived by MARXAN for individual benefit functions (individual networks) is displayed in Figure 3B. In most comparisons, the number of shared planning units is more than expected, but in some cases (i.e., with pollination and recreation, and pollination and forage production), there are far fewer shared prioritized planning units than expected. The same overlaps are shown as percentages of the smaller of the two relevant networks in Figure 3C. In many cases, these overlaps are considerable fractions of the smaller networks, exceeding 50% in several cases and 30% in most, so they are substantial in practical terms.

**Figure 4:**
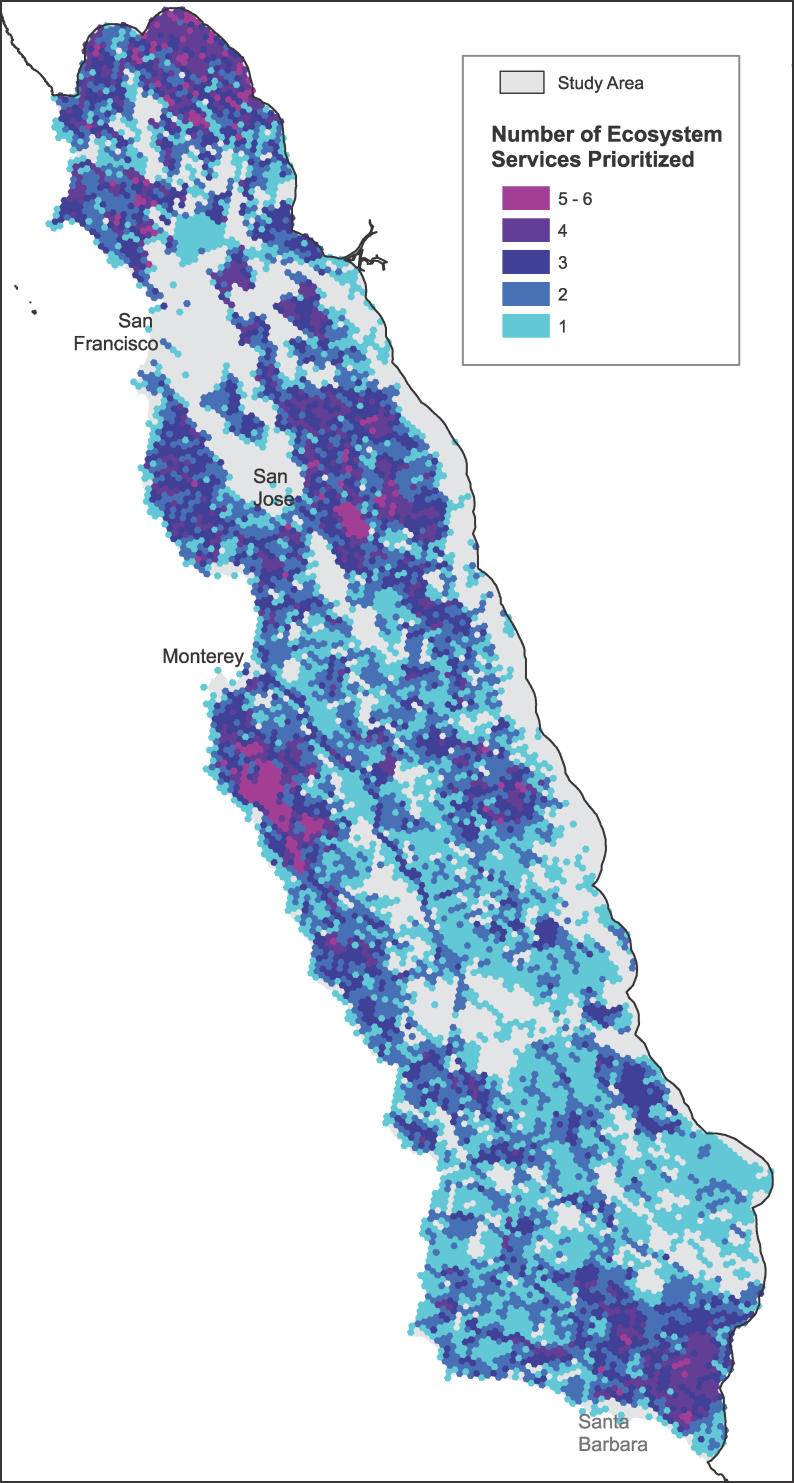
Ecosystem Service and Biodiversity Hotspots Colors represent the number of features for which each planning unit was selected in the individual-service best MARXAN network. We selected 1.8% of planning units for ≥5 features and 8.5% for ≥4.

The seven individual networks are summed over space in Figure 4, highlighting the distribution of areas selected for different numbers of benefits. Some highly urbanized areas are not selected by MARXAN for any benefits (e.g., San Francisco, San Jose, and the northwest corner of Santa Clara County, in gray), in part because we excluded highly developed lands from some service networks and in part because such lands simply do not provide high levels of services or are unsuitable for management for services.

Other areas, such as San Luis Obispo County, are selected for only a few benefits (in light blue). Although much of San Luis Obispo County is agricultural, there are few high-value crops that benefit from animal pollination. Furthermore, because of the sparse forest cover, this county has relatively low values for carbon storage. Although livestock values are relatively high in San Luis Obispo County, neighboring Kern County has far higher values likely because of nearby feedlots, slaughterhouses, and transportation routes. Accordingly, Kern dominates the network for forage production (Figure 2E). A portion of the planning units, for example in the northern Diablo Range (Figure 4, in pink), were selected for multiple benefits. The value of this area for carbon, water, and recreation is explained above. In addition, the relatively intact oak woodlands are important to biodiversity, forage production, and—owing to the proximity to dense population in San Jose—flood control. Interestingly, both this hotspot of overlap and the hotspot in the North Santa Lucia Range are areas where considerable public land has already been protected.

### Trade-offs

**Figure 5:**
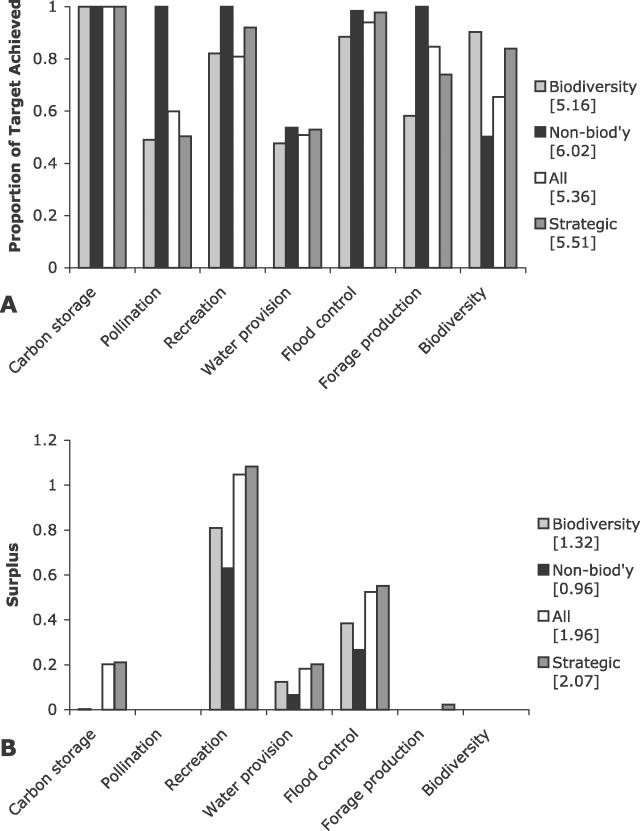
The Achievement of Alternative Strategies at Meeting Conservation Targets Target achievement is represented as the proportions of the seven targets achieved by four different conservation scenarios: Biodiversity (only biodiversity); Non-biodiversity (all except biodiversity); All; and Strategic (all but forage production and pollination: biodiversity, carbon storage, flood control, recreation, and water storage). (A) The average target achieved (achieved feature/target) across stratification units weighted by amount of target, capped at 1 where targets were exceeded. (B) The average amount by which targets were exceeded, weighted by target. The total target achievements and surpluses, summed across ecosystem services, appear enclosed in square brackets in the legend. This unweighted total underrepresents the contribution of biodiversity (which alone had hundreds of features, compared with one for each ecosystem service per stratification unit) to the planning process.

The extent to which individual benefit-function targets are met by the four comprehensive networks is depicted in Figure 5A: Biodiversity; Non-biodiversity; All; and Strategic (all except forage production and pollination, which removes all negative correlations and overlaps; see Methods). The “Biodiversity” network would protect a considerable supply of ecosystem services. All four networks achieve the carbon storage targets, but none achieves the water provision target (set at only 40% of total water use), and only one (“Non-biodiversity”) achieves more than 60% of the pollination target.

Because target achievement was assessed at the stratification-unit level and then aggregated to the ecoregion level, there are services for which there are considerable surpluses despite unmet targets (e.g., water provision; Figure 5B). In other cases, targets are well met, with surpluses (e.g., recreation, flood control) and without (e.g., carbon storage). The inability of “Non-biodiversity” and “All” to appropriately protect biodiversity demonstrates the risks to biodiversity associated with diluting the focus of conservation efforts without expanding the funds available for conservation. Such risks are greatly diminished when the ecosystem services targeted are chosen strategically (as in “Strategic”).

### Coincidence and Side Benefits

There are major differences in the extent to which benefit-function targets could be met through the biodiversity network alone or with additions. The pollination targets are only 49% met by biodiversity, but they only need 10% additional land (Table 2). This additional 10% contributes relatively little to biodiversity targets. Yet if protection or restoration of natural habitat adjacent to farms pays off entirely through pollination-augmented agricultural profits [13], these biodiversity benefits might come through strategic partnerships without the expenditure of conservation dollars.

**Table 2:**
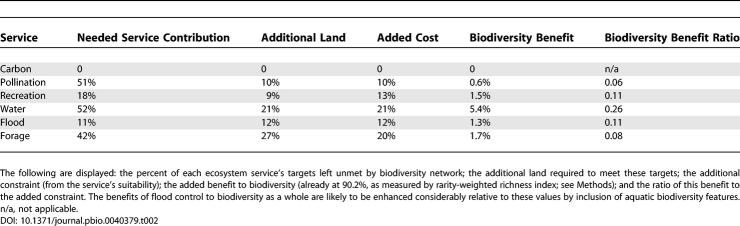
Results from Adding Individual Ecosystem Service Targets to the Existing Biodiversity Network

Contrast this situation with recreation, for which targets are 82% met by the biodiversity network. To achieve the remaining 18%, we need 9% additional land, which has far greater benefits for biodiversity. Because so much recreation would be provided by the biodiversity network, additional recreation funding could potentially contribute to conservation.

Carbon targets are met entirely by the biodiversity network because sites with high carbon storage are crucial for forest conservation. Carbon credits applied to forests in California would offer new funding for key elements of the biodiversity network, just as they offer promise in developing nations [85].

Finally, water provision targets are only 48% met by the biodiversity network, and the 21% additional land is highly valuable for biodiversity, both in total and per hectare. If this biodiversity value of lands valuable for water provision is a common phenomenon, great biodiversity benefits could accrue from the estimated 13% of terrestrial land that might be managed for urban water use [64].

### Key Insights

As human impacts on the environment expand in intensity and extent, there is a critical need to understand the degree of intersection between conservation priorities for biodiversity and for ecosystem services. This intersection of conservation priorities could achieve a measured and thoughtful balance between previously competing goals, while providing new sources of funding for its full-scale implementation.

The striking result of this preliminary analysis is the apparent contradiction between results of the spatial association and side benefit analyses. On the one hand are low correlations between the spatial distribution of the ecosystem-service benefit functions and relatively low levels of overlap between prioritized sites (Figure 3). However, despite the generally low correlations, there are hotspots where high values of multiple benefits coincide (Figure 4), although biodiversity protection was not strongly positively associated with any service (Figure 3). Protecting regions selected for their biodiversity value is not likely to maximize protection of the full suite of benefits unless there are considerable changes to the process by which biodiversity priorities are determined.

On the other hand, the biodiversity network would protect impressive supplies of ecosystem services (Figure 4). But networks configured to maximize the full suite of benefits could do even better (Figure 4). The relatively low overlaps between sites that are most appropriate for different features when prioritized separately do not negate the possibility of considerable gains from simultaneous prioritization: sub-optimal but valuable sites may coincide. Developing methodologies for such combined planning analyses should be a top research priority.

We adapt a general planning framework for biodiversity to planning for ecosystem services and do not present detailed representations of individual ecosystem services. A much deeper analysis is warranted. The coarse scale of the readily available data for many of the ecosystem services hinders analysis of ecosystem services. The carbon pool and pollination analyses require data with finer resolution within relevant boundaries (Figure 2C and 2D). For example, because most agricultural data are provided by political units such as counties, assessing pollination services requires interpolating fine-scale patterns from coarse-scale data, which likely introduces error. Despite the data limitations, this analysis yields five key insights for individual services, the relationships among them, and the exercise as a whole.

#### 1. Suitability and demand are determining factors.

As with biodiversity planning, the network design process for ecosystem services is strongly influenced by factors other than patterns of biophysical supply. Prioritized sites generally have high feature values, but two other factors determine planning unit selection: suitability (lower in urban areas) and targets (intended to represent demand and specific to stratification units, outlined in dark gray in Figure 2). High feature-value sites are not selected for two reasons: low suitability for conservation and low pertinent demand or need. For example, the site-selection algorithm did not select some sites of high forage production in Kings and Kern counties, whereas it did select some low–forage-production sites in San Luis Obispo and Fresno Counties. The former sites have lower suitability due to agriculture and urban development, whereas the latter are more remote.

Remoteness is relevant for water provision also, where targets are linked to actual water use. Here, several planning units in the Santa Cruz Mountains (which have high precipitation; stratification unit 5,000, Figure 2G) are not selected for water provision due to the relatively low demand compared to adjacent sites. Spatial mismatches between supply and demand complicate ecosystem-service provision and the planning for those services.

#### 2. Spatial scale.

Two important points pertain to the issue of spatial scale. First, benefits vary in the scale of their operation and dependence on habitat, and this may dramatically affect simultaneous management for multiple services. Most strikingly, biodiversity conservation generally requires large intact landscapes, but crop pollination arises from small patches of (semi-) natural habitat within a human-dominated landscape (we did not consider long-term sustainability of pollinators, which might require larger patches). Not surprisingly, the two features are negatively associated spatially (Figure 3), and each seems to greatly constrain the target achievement of the other in combined networks (Figure 5).

Second, independent scales of supply and demand can affect relationships between target achievement and the total size of benefit demand and supply. Targets may be poorly met despite relatively high overall availability or they may be well met despite barely adequate availability. Targets are more easily met if demand occurs at broad scales and supply varies considerably at local to regional scales. For example, carbon storage demand is global, but supply varies greatly based on vegetation cover and climatic conditions; consequently, it was possible to meet the target of 50% of the ecoregion's carbon storage in all networks (Figure 5). Although global demand makes it easier to meet regional targets, it also introduces artificiality: specific regional targets do not make much sense, because they ignore how well the global targets might be better met elsewhere. In contrast, when demand varies at smaller scales than supply, spatial mismatches are exacerbated and targets may be more difficult to achieve. For example, water demand accompanies agricultural use and residential development, which do not coincide spatially with areas of high water surpluses (precipitation minus evapotranspiration). Although water provision targets were easily met and exceeded in some stratification units (1,000, 5,000, and 6,000; see Figure 2G), they could not be met or even approached in others (2,000 and 3,000, even with a relatively low target of 40% of total water use).

#### 3. Population centers yield tensions.

For some ecosystem services, demand scales positively with the number of people in close proximity, whereas developed and agricultural lands are less productive or less suitable for management. These two factors result in a tension in planning, even for an individual service. For example, the demand and therefore the value of recreation opportunities is much greater close to cities (e.g., in the San Francisco Bay area, Figure 2F). When people have alternative sources of outdoor recreation (e.g., South Bay area, in and around San Jose, Figure 2F), the high value may be countered by low suitability (high costs of land management for recreation) such that high value sites are not selected by our method. When people have no other options (e.g., North of San Francisco, Figure 2F), however, the high value supersedes low suitability, and the planning units are prioritized. Similarly, flood control and water provision services are more needed near cities, but are generally degraded by development.

#### 4. Need new data, methods development.

To plan thoroughly for multiple ecosystem services, we need considerable advances in data and planning methodologies. Although there was sufficient data in this ecoregion for a first-pass analysis, planning for ecosystem services at smaller scales and in other ecoregions will likely require new research. For example, planning for crop pollination at finer scales requires an improved understanding of the contribution of individual pollinator species to particular crops, which is currently sparsely understood [86–88]. In other places, we anticipate that the kinds of data collected by relevant government agencies in California does not yet exist.

Although the application of MARXAN yielded insights, the tool lacks several features that are required for ecosystem-service planning. First, a new tool should allow a single network to include different features with different suitability layers. Specific suitability would reflect the factors that affect that particular feature's management. This would allow simultaneous planning for terrestrial and aquatic diversity.

Second, a new tool should incorporate the possibility that targets will not be met with available resources or that they may be met from outside the planning region. Third, an ideal tool would incorporate some spatial and temporal dynamics to account for the potential impacts of management and threats on species and services. Ideally, conservation would target areas for protection based on the potential for loss of benefits, not simply for the benefits supplied under current land use as in this analysis. Such dynamics should also allow the representation of the dependence of ecosystem functions on changes in biodiversity; although these effects might not be generally strong for ecosystem stocks and fluxes, they are likely more important for stability [89].

Fourth, such a tool should account for the fact that management for one purpose (e.g., threatened species) will be incompatible with management for another purpose (e.g., recreation). Fifth, a tool must account for the flow from particular ecosystems to particular beneficiaries. Site-selection software like MARXAN assigns value to a planning unit in the context of the larger stratification unit, without more specific accounting of spatial context or ecological processes [90]. For example, in modeling the contribution of natural vegetation cover to flood control, we accounted for the proximity to the floodplain and for the population density in the relevant watershed's floodplain, but we could not specifically account for the population downstream that would be directly impacted by flood mitigation.

Finally, a tool should allow flexibility between the ends of benefit maximization (used by Naidoo and Ricketts [91]) and suitability-maximizing target achievement (used here), which will each be appropriate for individual ecosystem services in different circumstances. Benefit maximization will be especially appropriate when services have substitutes whose appropriateness will also vary spatially; suitability maximization will be appropriate for features like biodiversity and perhaps recreation, for which the motivation for protection is principle rather than private preference [92]. Such flexibility will allow more effective analysis and the increased potential for engaging partners whose interests in the full suite of ecosystem services will differ.

#### 5. Need multidisciplinary and transdisciplinary teams.

Ecosystem-service planning must involve multidisciplinary and transdisciplinary teams. Interdisciplinarity (research between disciplines) is not sufficient, because ecosystem-service research and planning requires deep knowledge within—and across—multiple disciplines. Planning for ecosystem services requires expertise in biology, chemistry, physics, economics, finance, geosciences, geography, and particular analytical tools. The integration of theoretical understanding and empirical expertise from these diverse fields therefore requires a multidisciplinary team of experts working in close communication, spearheaded by transdisciplinary scholars and practitioners.

#### 6. Consider trade-offs and side benefits.

Only by analyzing both the trade-offs and the side benefits for biodiversity of conserving ecosystem services and vice versa can we guide conservation efforts more effectively. Trade-off analyses will be applied most successfully when management for an ecosystem service cannot help to meet the targets of biodiversity conservation.

Analyses of the ancillary benefits of an ecosystem-service project to biodiversity conservation and vice versa have two purposes. Such analyses can reveal when an ecosystem-service project offers promise for attracting new conservation partners and funds for biodiversity projects, and when such projects are especially important for their biodiversity benefits.

By combining trade-off and side-benefit analyses with a thorough scoping of potential partnerships and new markets, we may achieve substantial increases in biodiversity conservation while conserving the ecosystem services critical for human well-being. For example, case studies of water regulation and delivery and flood control reveal that conservation of forests and wetlands are sometimes worthwhile from an ecosystem-service perspective alone (in the Yangtze River watershed, China [14], around the Panama Canal [93], and in the Catskills and Charles River watersheds, US [94]). There are other places where such ecosystem-service values are undervalued or not quite sufficient to outweigh opportunity costs of conservation, but where the strategic investment of expertise and conservation funds could meet multiple goals simultaneously. If our results are representative of other places, lands for water provision and flood control may be particularly important for biodiversity conservation (Table 2).

### Conclusion

The inclusion of ecosystem services in conservation planning has great potential to provide opportunities for biodiversity protection. This preliminary exercise seems to suggests that conservation planning for other services—either separately or in combinations with biodiversity—may result in considerable declines in the ability to meet biodiversity conservation targets, but this finding stems from assuming no new opportunities. Furthermore, strategic choices of particular services to include in conservation planning can yield considerable gains. Our strategic network of five benefits—biodiversity, carbon, flood control, recreation, and water provision—eliminated negative associations between features. This “Strategic” network met targets far better than did the “All” benefits network, both overall and especially for biodiversity protection (Figure 5).

This study suggests that planning for ecosystem services would involve a major shift toward new geographies and a broadening of current conservation goals. The potential payoffs of such a shift are tremendous for both biodiversity conservation and human well-being [2,33,95,96], promising to sustain critical services, open new revenue streams, and make conservation broad based and commonplace. The goal of simultaneously maximizing biodiversity conservation and ecosystem services critical to poverty alleviation and general human well-being is one that can be embraced by all.

## Supporting Information

Protocol S1 Detailed Description of Methods. (124 KB DOC)
