# Supplementary material for: Conservation Planning for Ecosystem Services
Source: PLoS Biol. 2006 Oct 31;4(11):e379. doi: 10.1371/journal.pbio.0040379 (PMC1629036; doi:10.1371/journal.pbio.0040379)
Supplement: Supplementary file 1 [file pbio.0040379.sd001.doc]

## Protocol S1

### Biodiversity

The Nature Conservancy has updated its 1997 Central Coast Ecoregional Assessment by assembling spatial data on the distribution and viability of elements of biodiversity (conservation features) and using MARXAN v1.8.2 (Ball and Possingham 2000; Possingham et al. 2000) and expert input to delineate network conservation areas (The Nature Conservancy 1997, 2005). For biodiversity and the six ecosystem services the geographic extent of analysis included both the Central Coast Ecoregion and a 10 km buffer, to account for the contribution of immediately adjacent areas.

#### Feature Definition and Mapping

For the Central Coast Ecoregional Assessment update, we selected 546 conservation features to represent the Central Coast ecoregion’s biological diversity, including 89 coarse-filter (ecological systems based on dominant vegetation, abiotic aquatic modeled habitats) and 457 fine-filter (plant and animal species) features. For this analysis, we omitted the abiotic aquatic features because their representation in network conservation areas was evaluated after site-selection using watershed-based planning units and was not originally analyzed with the hexagonal planning units. We do not have ecological processes explicitly as conservation features, as processes can endure while biodiversity erodes (Groves et al. 2003). Of course, processes should be factored into an analysis of biodiversity priorities specifically as they impact the distribution and persistence of conservation features. The sections below discuss stratification, setting targets, and suitability, three factors that are used to ensure that conservation networks sustain ecological processes and flows.

#### Stratification

#### We delineated seven terrestrial stratification units to reflect major breaks in precipitation, temperature, vegetation, topography and related environmental gradients. Stratification is also used in recognition of the importance of the spatial dimensions of ecological processes, such as natural disturbance, gene flow, and wildlife dispersal. By ensuring redundancy and distribution across gradients, stratification helps address changes in these processes. Data sources included expert knowledge, review of USDA Forest Service Ecological Units of California (Miles and Goudey 1997), and review of statewide vegetation and climate maps (Davis et al. 1998; California Department of Conservation 2002; California Department of Fish and Game 2003).

#### Setting Conservation Targets

Our targets for ecological systems ranged from 40-100% of all mapped area, depending on spatial scale and rarity of the system. Using the NatureServe threat rankings (NatureServe 2005), the species-level targets had the following targets:

•	All viable occurrences for species that were “critically imperiled” or “imperiled” at the global (G1–G2) or subnational scales (S1-S2 for species and T1-T2 for subspecies)

•	50% of all viable occurrences (with a minimum of 24 occurrences total) for species and subspecies that were “vulnerable”, “apparently secure” or “demonstrably secure” (G3-G5, S3-S5, T3-T5) or unranked, with these occurrences stratified across their ranges (modified from Groves et al. 2003)

#### Suitability Layer (Planning Unit “Costs”)

The suitability index was created by calculating the weighted sum of four layers for each planning unit: weighted road density, developed land cover, agricultural land cover (not including grazing), and the length of all patch boundaries between natural or semi-natural land uses and urban or intensive agricultural uses. Individual cost factors were assigned a weighting based on our estimate of the relative severity of the factor to the integrity of conservation areas (see table below).

| **Layer Name** | **Weighting** | **Potential Max. Value** | **Data source** |
| --- | --- | --- | --- |
| Developed Areas | 4x | 2000 | CA Department of Conservation 2002 |
| Road Density | 2x | 1000 | Topologically Integrated Geographic Encoding and Referencing (TIGER) data (U.S. Census Bureau 2000) |
| Agricultural Areas | 1x | 500 | CA Department of Conservation 2002 |
| Non-natural edge | 1x | 500 | CA Department of Conservation 2002 |

MARXAN runs that used this suitability layer also excluded planning units from the network if they contained >50% developed or >80% agricultural cover.

### Carbon Storage

#### Feature Definition and Mapping

As our feature value for this service, we used a dataset developed by the USGS and Department of Energy (DOE) and further refined by World Resources Institute (WRI) (Loveland et al. 2000; Matthews et al. 2000). It provides an estimate of total (realized) carbon stored in above- and below-ground vegetation (tons/ha) for each grid cell of 82.81 km^2^. Given the spatial mismatch between the 500 ha planning units and the 83 km^2^ cells for carbon, we assigned the feature value to individual planning units by using the value that covered the majority of the hexagon.

#### Setting Conservation Targets

Because a realistic feature for carbon storage would have to take into account the anthropogenic emissions of carbon at a global scale, we set a feature based solely on what is available to store in the ecoregion, namely 50% of the carbon in above- and below-ground vegetation.

### Crop Pollination

#### Feature Definition and Mapping

Through literature (Free 1993; Delaplane and Mayer 2000) and expert input we generated a list of crops requiring or benefiting significantly from animal pollination. We summed the total economic value of these crops at the county level (excluding counties primarily within the Central Valley, as the crop land in these counties is almost entirely outside our ecoregion). This coarse county-scale data accounts for the fact that crop types grown will change considerably on the temporal scale of conservation within a given region, but it artificially ties this real geographic effect to political boundaries. To create the scaling factor used to weight agricultural areas where high value crops are animal pollinated, we divided the county’s total dollar value for animal-pollinated crops (in 2003, using data from county crop reports and consultation with county agricultural commissioners) by the total agricultural land area in each county that falls within the Central Coast ecoregion. To obtain a planning unit’s pollination value, we multiplied this scaling factor by a function of the land cover in that planning unit (percent of agricultural land + 0.25*percent in natural land cover—for units with some agricultural cover) (California Department of Conservation 2002). This relative weighting of land cover types reflects our assumption that pollination values at the small scale are determined more by the economic and biophysical factors that determine where farmers grow high-value animal pollinated crops than by the current presence of natural habitat (which can be restored).

Because it is unclear which insect species pollinate which crops, and because foraging distances (e.g., from refuges in natural vegetation to crops) vary over several orders of magnitude (Greenleaf 2005), we did not attempt to model the spatial configuration of crops and natural areas precisely.

As noted in the methods section, our benefit function does not require natural cover to exist currently in a planning unit in order for that unit to have a non-zero feature value. This assumption of possible habitat restoration impacts the association with other ecosystem services and biodiversity: e.g., planning units with >150 ha natural cover have considerably higher average biodiversity scores (by RWRI; see methods) than planning units with <150 ha natural cover.

### Flood Control

#### Feature Definition and Mapping

Our flood model attempts to capture the flood prevention and mitigation capacity of upland terrestrial habitats, addressing river flooding and not the ocean-inundation of coastal areas. A planning unit’s flood-control score is a simple additive function of four factors: the percent of natural cover in a planning unit, the percent of natural cover within the riparian zone of streams and rivers, the distance (following drainage routes) from a planning unit to the 100-year flood plain, and the percent of land in agricultural cover. We used the Farmland Mapping and Monitoring Program (FMMP) (California Department of Conservation 2002) data for developed, agricultural, and natural cover. Distance to floodplain was calculated using the FLOWACCUMULATION function in Arc/Grid (ESRI 2004) and assigning a value to each planning unit corresponding to that planning unit’s share of the average maximum flow accumulation in the watershed’s floodplain. Due to significant data gaps in the National Wetlands Inventory we were unable to include wetlands in our model, despite the importance of their contribution to flood mitigation (U.S. Fish and Wildlife Service 2004). As with other services, we modeled only the core elements of the service and did not address the existing dams, levees and other flood control structures in place.

#### Stratification

We chose stratification units commensurate with the scale at which flood risk can be mitigated, in watersheds. We used watershed-based Hydrologic Unit (HU) boundaries from a California watershed database, Calwater 2.2a (California Department of Water Resources 2003). We clipped the watershed boundaries on the east side of the ecoregion to the 10-kilometer buffer. We aggregated several watersheds in the San Joaquin River, Tulare Basin, and Sacramento River basins because their floodplains are outside the ecoregion and individually their population densities and distances to floodplain underestimate their importance to the rest of the system to the east. After these aggregations, there were 25 stratification units (Figure 2).

#### Setting Conservation Targets

Using U.S. Census data—modified to account for the lack of people living on public land (California Department of Forestry and Fire Protection 2003)—we calculated the total number of housing units in those census blocks with their centroid within the 100-year floodplain as mapped by FEMA (Federal Emergency Management Agency 1995). Our targets for the flood control network were based on these housing-unit numbers for each stratification unit divided into quantiles, as described below.

| **Quantile** | **Min # of Housing Units in Stratification Unit** | **Target: % of Total Service Value in Stratification Unit** |
| --- | --- | --- |
| 1 | 14109 | 80 |
| 2 | 7036 | 60 |
| 3 | 1343 | 40 |
| 4 | 533 | 20 |
| 5 | 1 | 10 |

### Forage Production

#### Feature Definition and Mapping

Our approach to modeling forage production service value was similar to that used for crop pollination: we scaled the amount of forage produced by the economic value of that forage to the production of livestock. We used the average range-production value for each map unit in the SSURGO county soil survey data (Natural Resources Conservation Service 2002). This measured variable—based on clipping and weighing of dry herbaceous biomass—is the “representative” range-production value for each map component (Curtis Talbot, NRCS, personal communication). To obtain the total range-production value of a planning unit, we multiplied the average range-production value in that planning unit by the percent of land in cover types that could support rangeland livestock (grassland or oak woodland, as mapped in the vegetation systems data used in the Ecoregional Plan update).

In order to represent the economic significance of forage production, we scaled the modified range-production values (described above) by the economic-value-intensity of rangeland. This scaling factor was calculated at the county level by dividing the total dollar value of rangeland livestock/products by the county’s total range production value. The value of rangeland livestock and products was calculated from county agricultural commissioner reports (2003) and consultation with county agricultural commissioners. Those counties that are not completely within the study area had to be re-scaled to account for the economic value derived from the portion of the county in the study area. The simplest calculation would have been to multiply the percentage of the county’s rangeland production value in the study area by the county’s total economic value. Unfortunately, this would have been overly coarse: the counties on the eastern border of the study area have distinctly different vegetation types and land uses in the study area than in Central Valley. To account for this, we obtained additional information from county agricultural commissioner staff in the form of estimated percentages of total livestock values in the county that stemmed from the Central Coast ecoregion.

#### Stratification

Because of the presumed synergies between biodiversity and forage production and the need to disperse extractive land uses like livestock grazing, we used the same stratification and cost factors as with biodiversity.

### Recreation

#### Feature Definition and Mapping

In modeling recreation service, we made several assumptions: natural or semi-natural landscapes are desirable; areas near large population centers will be more heavily used than remote landscapes; proximity to major roads increases the recreation use of an area; and publicly accessible land is more important for recreation than private land. Following the first assumption, we adjusted the recreation-service value by multiplying the sum of the feature value by the amount of land with natural or agricultural cover. Below we explain how we addressed the other three assumptions.

Proximity to Population Centers:

We used the same modified U.S. Census (2000) block-level population data described under the flood control section (California Department of Forestry and Fire Protection 2003). We created a population density surface by using a moving window analysis to sum the population density of blocks that have their centers within ten kilometers of each cell in a grid at a resolution of 25 ha. We used density and not total number of people because the range of sizes of census blocks is very large, and values from large blocks that are not area-adjusted could misrepresent where people live. We classified this population density surface into 10 natural breaks using the Jenks optimization and then weighted these values ranging from 1 to 40 (Jenks 1967).

| **Population Density Sum** | **Natural Breaks** | **Weighting** |
| --- | --- | --- |
| 209618 | 1 | 1 |
| 295422 | 2 | 1 |
| 707279 | 3 | 1 |
| 1076234 | 4 | 5 |
| 1488092 | 5 | 10 |
| 2037235 | 6 | 15 |
| 2912432 | 7 | 20 |
| 3873432 | 8 | 30 |
| 4611343 | 9 | 40 |
| 16463126 | 10 | 40 |

Distance to Major Roads:

We used a major roads layer for the state that was developed from Topologically Integrated Geographic Encoding and Referencing (TIGER) data (U.S. Census Bureau 2000). We assumed that larger roads provide recreational access to more people for recreation, and therefore support higher service values. In order to account for this, we calculated the distance to road buffers using buffers that vary by road type.

| **Road Type** | **Buffer Width (m)** |
| --- | --- |
| Expressways | 2000 |
| Primary roads | 500 |
| Secondary and connecting roads | 250 |

We represented the distance to those road buffers by values ranging from 1 to 10 based on the square root of the distance, with areas near roads receiving higher scores than those far from roads.

Public Access:

One of the most significant factors in determining an area’s value for recreation is the extent to which it can be accessed legally by the public. We classified our land ownership data into three classes and, in the absence of necessary data, assigned arbitrary weights: (1) areas with no public access (e.g. private lands; weight = 0), (2) areas with restricted access (e.g. private conservation land, watershed lands; weight = 5) and (3) areas with full public access (e.g. national forests; weight = 30). The particular weights are somewhat arbitrary, but we had no data on the recreation use of private or restricted lands. Military lands were excluded for this service.

#### Stratification

We chose stratification units to ensure recreation services within a reasonable distance of population centers. Units were defined by aggregating counties around major urban centers and along latitudinal gradients. This is a simplification of actual patterns of recreation, because people do cross boundaries—of stratification units and the ecoregion—in seeking recreation opportunities (e.g., coastal areas provide important recreational opportunities for people living in the Central Valley, to the east).

#### Suitability Layer

Based on the assumption that intact natural areas provide higher recreation values than modified or degraded areas, we used the same suitability layer as the biodiversity analysis.

#### Setting Conservation Targets

We set a target of providing sufficient land in each stratification unit to meet the recreation demands for each person, assuming an average demand of 12 recreation days per person per year (one per month). Clearly, the amount of land necessary depends in part on the appropriateness of the contributing areas for recreation. We transformed our feature value to obtain that metric of appropriateness or “effectiveness”. We derived a baseline estimate for the amount of land needed per person from the actual usage of an ideal case, Golden Gate National Recreation Area (GGNRA). GGNRA is a national park in a relatively natural state, but very close to San Francisco and represents a heavily used, managed open space tract. We calculated the amount of space needed per visitor-day by dividing the average number of visitors per day to GGNRA by the acreage of the park. There were 13,270,547 visitor days in 2004 in the 30,506 hectare park, yielding a per-visit land area of 0.0023 hectares (National Park Service 2004).

We translated the original feature values (from 1 to 80) to “effective hectares” by dividing by the maximum (80) to obtain an “effectiveness proportion”. We then multiplied this proportion by the land area of the hexagon (in ha). For example, a planning unit with a mean feature value of 80 would contribute 500 effective hectares toward the target ((80/80 effectiveness) * 500 ha = 500 effective ha); a planning unit with a mean of 40 would contribute 250 effective hectares ((40/80 effectiveness) * 500 ha = 250 effective ha).

### Water Provision

#### Feature Definition and Mapping

Our model was a function of net precipitation (precipitation minus evapotranspiration) as calculated by monthly averages from 1996-2000 with a spatial resolution of 1 km. The precipitation data layer was developed at the NASA Ames Research Station by interpolating climate station data of the National Climatic Data Center (NCDC) and Climate Prediction Center (CPC) using an inverse distance weighting method. Evapotranspiration data were obtained using the Terrestrial Observation and Prediction System (TOPS) (Nemani et al. 2003; White and Nemani 2004). The inputs to this model were temperature, precipitation, vapor pressure deficit, and surface radiation, as calculated by the methods of Jolly et al. (2005), and the eight-day composite MODIS products of photosynthetically active radiation absorbed by plant canopies (FPAR) and leaf area index (LAI) (MOD15 collection 4; Myneni et al. 2002).

We translated the original precipitation and evapotranspiration data (units of mm/day for each month) into average annual net precipitation. To account for the degradation of water quality from urbanized areas, we multiplied the average net precipitation value per hexagon by the proportion of land not in developed cover (California Department of Conservation 2002) and excluded hexagons with greater than 90% developed cover. We also excluded coastal watersheds of first order streams that drain directly into the ocean, since water falling in these watersheds would be largely unavailable to human use.

#### Stratification

We used the same stratification units as the biodiversity analysis, which are loosely based on large-scale watersheds. We chose watersheds of a large scale in recognition of leakage between watersheds (Hayward 2005) and the human propensity to divert water from other watersheds, particularly adjacent ones.

#### Setting Conservation Targets

We based our targets for this service on actual water use, using county-level statistics for 2000 (U.S. Geological Survey 2000). We included all freshwater use (ground and surface) for residential, agricultural, and industrial purposes. We summed water usage by stratification units by taking the proportion of a county’s developed and agricultural land in the stratification unit and multiplying the county usage total by this proportion. Given the considerable amount of water delivered to our ecoregion from the Sierra Nevada Mountains by pipes, aqueducts, and more natural flow, we set targets of 40% of total use for each stratification unit.

***MARXAN Settings***

There are many settings and values that can be adjusted in MARXAN that affect the scenario results. Because the focus of this paper is to analyze the tradeoffs of planning for biodiversity and ecosystem services, we wanted to minimize artifacts of the algorithm in selecting planning units to assemble a portfolio. The same settings were used in MARXAN scenarios whenever possible for the individual service scenarios and the four combined scenarios. The only setting that we altered was the species penalty factor, as these were adaptively set for each service based on the minimum penalty that would ensure that targets were met.

The settings that were used in all of the individual runs (biodiversity and six ecosystem service runs) are as follows:

Boundary length modifier: 0.0001

Clumping: rising benefit function

Algorithm used: annealing and iterative improvement

Heuristic used: none

Number of iterations: 1000000

Initial temperature: 10000.00

Cooling factor: 0.980000

Number of temperature decreases: 10000

Cost threshold: disabled

Threshold penalty factor A: N/A

Threshold penalty factor B: N/A

Random Seed: -1

Number of runs: 20

The values for species penalty factors were as follows:

Biodiversity: ranged from 1-5

Carbon storage: 1.7

Crop pollination: 2

Forage production: 3

Flood control: 1.3

Recreation: 2

Water provision: 1.5

## References
